# Do intensified job demands predict burnout? How motivation to lead and leadership status may have a moderating effect

**DOI:** 10.3389/fpsyg.2023.1048487

**Published:** 2023-03-09

**Authors:** Katariina Lehtiniemi, Anni Tossavainen, Elina Auvinen, Mari Herttalampi, Taru Feldt

**Affiliations:** Department of Psychology, Faculty of Education and Psychology, University of Jyväskylä, Jyväskylä, Central Finland, Finland

**Keywords:** intensified job demands, intensified learning demands, occupational well-being, affective-identity motivation to lead, resources, sustainable careers, burnout

## Abstract

**Objectives:**

The aim of this longitudinal study was to investigate how intensified job demands (job-related planning demands, career-related planning demands, and learning demands) are associated with burnout. We explored whether affective-identity motivation to lead moderates this association and, thus, functions as a personal resource regardless of leadership status. We further investigated whether the possible buffering effect is stronger for those professionals who became leaders during the follow-up.

**Methods:**

Our sample consisted of highly educated Finnish professionals (*n* = 372): part of them (*n* = 63, 17%) occupied a leadership position during the 2-year follow-up while the rest maintained their position without formal leadership duties.

**Results:**

The results of hierarchical linear modeling indicated that intensified learning demands were associated with later burnout. High affective-identity motivation to lead was not found to buffer against the negative effects of intensified job demands - instead, it strengthened the connection of intensified job- and career-related demands to burnout. Nevertheless, among the whole sample, professionals with high affective-identity motivation to lead reported lower burnout when job demands were not highly intensified. The leadership status also played a role: High affective-identity motivation to lead strengthened the connection of career-related demands to burnout in those professionals who became leaders during the follow-up.

**Conclusions:**

Altogether, we propose that in certain circumstances, affective-identity motivation to lead might help professionals, with and without formal leadership duties, to be more ready to lead their own work and well-being. However, in order to promote sustainable careers, the vulnerability role of high affective-identity motivation to lead should be considered as well.

## 1. Introduction

Professionals, i.e., knowledge-workers, in modern society are impacted by new ways of working and intensified job demands (IJDs); they are increasingly required to make key decisions, adopt new skills and knowledge, and plan their own jobs and careers (Kubicek et al., 2015; Kotera and Vione, 2020). Leadership is typically awarded to highly educated professionals for good work: when professionals occupy leadership positions it brings another dimension of high demands (Skakon et al., 2011; Li et al., 2018). General accelerated pace of life (Rosa, 2003, 2013), IJDs (Kubicek et al., 2015), and, for example, the fact that employees are required to manage their own work ability, might obscure one’s own boundaries, the borders of adequate performance level, and the perception of what is normal within the spectrum of occupational well-being. If job demands are long-term and constantly exceeding a person’s limited resources, they can pose a risk for occupational well-being (Bakker and Demerouti, 2017; Mauno et al., 2022). Baruch and Vardi (2016) have proposed that such modern workplace factors as career self-management may indeed risk individuals’ well-being and sustainable career development. People in the modern workplace are adapting to change and proactively shaping their career as agents (De Vos et al., 2020). For example, not all professionals “drive” toward leader positions –some “drift” toward them (Auvinen et al., 2021). Career choices that fit one’s own personal values are more likely to provide a sustainable career (De Vos et al., 2020). The framework of sustainable careers (De Vos et al., 2020) integrates the dimensions of person, context, and time to explain the complexity of career paths; meanwhile the indicators of sustainable careers –happiness, productivity, and health – also form a basis for individual, organizational, and societal welfare (De Vos et al., 2020).

The motivation to lead (MTL; Chan and Drasgow, 2001) describes both a stable and dynamic difference between people in terms of the decisions they take about whether to pursue further training and assume greater leadership roles and responsibilities. MTL is generally considered to be a resource that supports more sustainable careers for leaders because it seems to promote occupational well-being (Auvinen et al., 2020; De Vos et al., 2020). However, while we agree that MTL is certainly a useful resource for highly educated professionals wanting to pursue a leadership position later in their career, we must remember that leadership-related tasks often fall also on those not in formal positions of leadership (Yukl, 1989; Bass and Bass, 2008). In present-day work environments, for example, self-leadership, entrepreneurial behavior, and shared leadership are increasingly valued within organizations as they are often associated with better performance (Zhu et al., 2018; Goldsby et al., 2021; Pirhadi and Feyzbakhsh, 2021). Professionals are also expected to be increasingly self-directed and flexible in managing both their career development and daily work activities (Kubicek et al., 2015), regardless of their position. Leadership could thus be seen as part of all kinds of professional work. Certainly, there are differences between formal and informal leader roles. However, it could be assumed that those professionals who find leadership interesting and motivating could more easily find sources of meaningfulness when working in previously mentioned environments.

Indeed, this study has an explorative perspective regarding the role of ‘affective-identity motivation to lead’ (AI-MTL) for professionals without formal leadership status. As the borders between professional and formal leader positions are not as clear as they were in the past, we wanted to pursue the possibility to gain novel knowledge regarding AI-MTL as a resource for highly educated professionals. In addition, we chose to study AI-MTL as our sample also consists of those professionals who occupy formal leadership positions during the 2 year follow-up. Research on MTL as a resource is scarce overall (see Auvinen et al., 2020, 2021) and non-existent for those professionals without formal leadership status. We are interested to see whether AI-MTL acts as a buffer against the negative effects of IJDs on occupational well-being, and to note any differences during the follow-up in the ways AI-MTL affect those in leadership positions and those who are not; is it a personal resource that might buffer against negative well-being outcomes regardless of leadership status? Beyond MTL research, white-collar workers have reported more intensification in planning, decision-making, and learning demands compared to, for example, blue-collar workers (Mauno et al., 2020). Thus, investigating resources for this group is particularly relevant. Moreover, as job demands are unlikely to decrease in the future, exploring resources such as AI-MTL also in non-leadership contexts would help individuals and organizations not only cope but actually thrive –while creating more sustainable careers. In the following chapters, we will introduce the main constructs and theoretical models of our study as well as the detailed hypotheses that will be tested.

## 2. Literature review

### 2.1. Occupational well-being as an indicator of career sustainability and intensified job demands (IJDs)

IJDs, introduced by Kubicek et al. (2015), are thought to have five dimensions: work intensification (WI); intensified job-related planning demands (IJPDs); intensified career-related planning demands (ICPDs); intensified knowledge-related learning demands (IKLDs); and intensified skill-related learning demands (ISLDs). However, because IKLDs and ISLDs overlap both conceptually and empirically, there are previous studies (e.g., Mauno et al., 2020; Huhtala et al., 2021) which have combined them as *intensified learning demands* (ILDs). This study will look at three of the above-mentioned aspects: (a) IJPDs, referring to the need employees increasingly feel to organize their work autonomously, set goals, and monitor the end results. (b) ICPDs, referring to the increasing responsibility they feel to form networks and monitor career prospects. and (c) ILDs, referring to the expectation that employees should constantly revise and renew their job-related knowledge and skills at work. There were two reasons for not including WI in this study: firstly, the three aspects chosen all concern *career* pressures –the focus of this study –whereas WI is about pressures felt more generally with regard to the working day (Kubicek et al., 2015); secondly, these three aspects have overall been less studied than WI (Mauno et al., 2019).

In the present study we investigate occupational well-being (i.e., ‘low burnout’) as an indicator of sustainable careers which reflects the health dimension of the model (De Vos et al., 2020). Burnout is defined as a psychological syndrome emerging as a response to prolonged, chronic, work-related stressors (Maslach and Jackson, 1981; Maslach et al., 2001). It manifests itself as exhaustion – feeling that emotional and physical resources are being depleted; cynicism – feeling negative and distant toward different aspects of work; and reduced professional efficacy – feeling a lack of achievement, productivity, and competence at the job (Maslach et al., 2001). We refer to reduced professional efficacy in this study as inadequacy (Salmela-Aro et al., 2011; Feldt et al., 2014). The consequences of burnout are widely recognized both on the individual level –as psychological and physical health problems, and organizational level –e.g., employee absenteeism (Salvagioni et al., 2017). In fact, burnout can be described as ‘an enduring psychological condition of ill-being signaling that employees are no longer able and no longer willing to invest effort in their work’ (Bakker and De Vries, 2021, p. 3). In addition to the humanitarian cost for organizations and society, there is also an economic one (Maslach and Leiter, 2017), so burnout prevention is a crucial issue for every employer.

The Job Demands-Resources model (JD-R) categorizes work conditions into job demands and job resources (Demerouti et al., 2001). Although the name would indicate that the model refers exclusively to job resources (e.g., autonomy), in more recent studies personal resources (e.g., self-efficacy) have been included as well (Bakker and Demerouti, 2017). Both these kinds of resources can act as a buffer and become particularly important when job demands intensify (Bakker and Demerouti, 2017). To some extent, it is advisable to encourage learning at work, along with the improvement of competencies, and greater self-directivity (e.g., Van der Heijden and Spurk, 2019), but the resulting IJDs might also compromise occupational well-being. IJDs have, for instance, been found to associate with higher cognitive stress symptoms (Rantanen et al., 2021) which may eventually lead to burnout (Maslach et al., 2001), and one cross-sectional study among Finnish employees found that IJPDs, ICPDs, and ILDs all relate to higher overall burnout (Mauno et al., 2019). Meanwhile, another cross-sectional study on Finnish health care staff also found that IJPDs, ICPDs, and ILDs positively associated with higher exhaustion, but not with higher cynicism (Huhtala et al., 2021).

Since job demands can be seen as either hindrances or challenges (Cavanaugh et al., 2000; Mauno et al., 2022), some professionals might feel motivated by the challenge to increasingly self-direct their work, while others might be distressed by the hindrance. However, meta-analyzes (Crawford et al., 2010; Mazzola and Disselhorst, 2019) show that higher demands –regardless of their type –are generally linked to adverse health outcomes (including burnout). Overall, there is strong evidence on job demands triggering a health-impairment process (Bakker and Demerouti, 2017). IJDs are thus likely to lead to burnout and reduce occupational well-being, whether or not there are personal resources to mitigate this relationship. Previous studies thus brought us to our first hypothesis:

*H1:* High IJPDs (H1a), ICPDs (H1b), and ILDs (H1c) among highly educated professionals relate to higher burnout (exhaustion, cynicism, and inadequacy) 2 years later.

### 2.2. Motivation to lead (MTL) as a personal resource

Bakker and De Vries (2021) have most recently introduced self-regulation perspectives to the JD-R model, pointing out that different personal resources are key to how employees can prevent and reduce burnout (from high job demands and low resources) by influencing their own job characteristics through self-regulation strategies. Since differences between individuals affect how environments are interpreted, the fit between employees and their working environment should be more widely recognized when preventing burnout. From the career-development perspective of this study, this is particularly pertinent because the focus is on person-career fit, and a good person-career fit is more likely when career decisions are made according to one’s own personal values (De Vos et al., 2020). If the work captures the intrinsic value of contributing to a broader purpose as well as self-actualization, it is likely to be perceived as more meaningful and contribute to one’s well-being (Martela and Pessi, 2018). This intrinsic motivation can thus work as a personal resource and build up resilience to job demands.

AI-MTL is one way to conceptualize intrinsic motivation. It is part of a three-dimensional model of MTL (Chan and Drasgow, 2001), referring to leading out of joy. The other two dimensions refer to leading based on duty or responsibility (social-normative motivation) and leading without calculating the costs and benefits regarding the leadership position (non-calculative motivation) (Chan and Drasgow, 2001). Although the weight is on intrinsic motivation and a natural, personality-related inclination to lead, Chan and Drasgow (2001) do not assume AI-MTL to be an inborn quality; they suggest it can also be learned and reinforced by supporting self-efficacy *via* feedback in leadership training (Chan and Drasgow, 2001; Badura et al., 2020). In the present study, we examine AI-MTL as a personal resource for coping with IJDs and as a buffer against burnout. Hitherto, the only study concerning the relationship between MTL and occupational well-being is a cross-sectional study conducted among leaders (*n* = 1,003) by Auvinen et al. (2020). Occupational well-being (i.e., low burnout and high work engagement) was found to be highest among those leaders who reported high AI-MTL, suggesting that AI-MTL can be seen as a personal resource among leaders and highlighting the importance of person-career fit (Auvinen et al., 2020). In this study, we aim to provide a novel perspective by studying AI-MTL as a resource (or moderator) among highly educated professionals whose status may change from a professional to a formal leader during the study’s follow-up.

Compared to other types of MTL, AI-MTL has been linked to higher agentic orientation (Badura et al., 2020; Auvinen et al., 2021) –implying the tendency to be, for example, assertive, confident, ambitious, and independent (Eagly and Karau, 2002). One could suggest that people with these kinds of tendencies are also more daring to be in charge and pursue career-related responsibilities. Those professionals could find it more meaningful to work in environments that require skills and qualities related to leadership (e.g., self-leadership, entrepreneurial behavior within an organization, shared leadership) who have the following qualities in terms of their personality and values: (1) have a genuine interest in leadership and experience an intrinsic desire (or need) to lead. (2) find it comfortable to take the lead in a team, and (3) perceive themselves to be contributing more as a leader than they would as a follower (Chan and Drasgow, 2001). Having a high AI-MTL may promote their person-career fit (De Vos et al., 2020), whereas those professionals who do not find leadership meaningful, might feel more easily overwhelmed about the part of professional work that requires qualities related to leadership. Additionally, proactive personality has been cited as an efficient personal resource, as it allows employees to recognize and regulate the strain they experience (Bakker and De Vries, 2021). Such proactiveness also describes the agentic orientation which, again, has been associated with AI-MTL (Badura et al., 2020). These are examples of how AI-MTL might help to cope with demands that relate to planning, decision-making, and learning, whether or not one is a leader. This led us to our second hypothesis:

*H2:* AI-MTL moderates the relationship between the three IJDs and burnout: high AI-MTL buffer against the negative effects of IJPDs (H2a), ICPDs (H2b), and ILDs (H2c) on later burnout.

### 2.3. The importance of affective-identity motivation to lead (AI-MTL) for professional career paths

As formal leadership is typically awarded to highly educated professional for good work, it is possible that they occupy these positions for external motives (Chan and Drasgow, 2001); for instance, they might take the role out of a sense of duty, or for the economic advantages it offers (e.g., Auvinen et al., 2021). If a leadership position is accepted when the motivational resources to lead are low or non-existent, occupational well-being and career sustainability may suffer, due to a poor person-career fit and a sense of meaninglessness (Auvinen et al., 2020; De Vos et al., 2020). We expect AI-MTL to serve as a personal resource for highly educated professionals both with and without formal leadership roles considering the requirements of contemporary careers (as previously discussed). However, there are three reasons we assume that the buffering effect against burnout will be stronger among those who become formal leaders during the follow-up.

One reason is because the focus of AI-MTL has been to identify and develop motivated and high-performing candidates to fill leadership roles (Chan and Drasgow, 2001). This means the concept is probably better at capturing the specific elements of *formal* leadership positions than of those positions where leadership-related qualities also happen to be useful, such as professionals who are their own bosses or share leadership with their peers. Secondly, given our sample contains those who change their status to a leader, there are some special features that need to be considered. Previous studies have noted that employees experience more IJDs when their situation at work changes (Kubicek et al., 2015), so motivational resources would become particularly relevant for those who become leaders. Lastly, on top of the general intensification in demands at work (Kubicek et al., 2015; Mauno et al., 2019), leadership positions in particular are associated with high demands (Skakon et al., 2011; Li et al., 2018). Coping with these demands might be challenging if a leader has weak AI-MTL, as leaders with higher AI-MTL are more likely to believe in their leadership capabilities (i.e., leadership self-efficacy; Chan and Drasgow, 2001; Badura et al., 2020). Experiences of self-efficacy may help them feel in control even if aspects of work are indeed demanding and stressful. Not experiencing leadership self-efficacy could lead to burnout among professionals in formal leadership roles more easily than others, since weaker AI-MTL would be less important for those whose primary task is not leadership. Our third hypothesis is therefore:

*H3:* The moderating (buffering) effect of AI-MTL in the context of high IJPDs (H3a), ICPDs (H3b), and ILDs (H3c) is stronger among those who occupy a leadership position during the follow-up compared to those who maintain their professional position.

## 3. Method

### 3.1. Participants

The original sample of this longitudinal study was drawn from the membership registers of four Finnish trade unions in March–April 2017 (T1): the Finnish Union of University Professors, the Finnish Union of University Researchers and Teachers, the Finnish Business School Graduates, and the Academic Architects and Engineers in Finland (TEK). An electronic survey was sent to all members of the first two trade unions mentioned here (excluding those who have retired), and an electronic survey was sent to a random sample of 3,000 members of the latter two unions. In total, an electronic survey was sent to 9,998 union members of which 2,200 responded (response rate 22%). Two years later, in March–April 2019 (T2), the follow-up survey was sent to those participants who had participated in the baseline measurement and had given us their permission to include them in the follow-up (*n* = 1,013). The total number of these participants who responded in the follow-up was 694 (response rate 69%).

The sample used for the present study comprised those participants who were not already working as leaders at T1 and who also participated in the follow-up survey at T2 (*n* = 372). These participants either maintained their original professional position without leadership duties (*n* = 309, 83%) or had moved on to a leadership position (*n* = 63, 17%) 2 years later. The sample consisted of slightly more women (*n* = 220, 59%) than men (*n* = 152, 41%); the age range of participants was 25–66 years (*M* = 44, *SD* = 9.95); and the hours they worked per week varied between 5 and 75 h (*M* = 41.33, *SD* = 7.06). There were 12 professors (3%), 204 other university academics and researchers (55%), 71 business school graduates (19%), and 85 technical academics (23%) in the sample at T1.

### 3.2. Measures

*Intensified job demands (IJDs)* were measured using the Intensification of Job Demands Scale (IJDS; Kubicek et al., 2015) at T1. Participants were asked to assess possible changes they had experienced at work during the last 5 years (or during their whole time in the job, if they had been in the organization less than 5 years). Knowledge-and skill-related demands were combined since their mean scores were highly correlated (*r = *0.82, *p < *0.001) –resulting in three subscales: *intensified job-related planning demands (IJPDs)*, which had 5 items, e.g., ‘one increasingly has to check independently whether the work goals have been reached’ (*M* = 3.59, *SD* = 0.96, *α* = 0.86); *intensified career-related planning demands (ICPDs)*, which had 3 items, e.g., ‘one increasingly has to plan one’s professional career independently’ (*M* = 3.76, *SD* = 0.95, *α* = 0.79); and *intensified learning demands (ILDs)*, which had 6 items, e.g., ‘one has to acquire new expertise for the job more often’ (*M* = 3.65, *SD* = 0.94, *α* = 0.93). Answers to each item were given on a 5-point Likert scale (where 1 = not at all and 5 = completely). From these, the mean scores were then calculated for the three dimensions with higher scores indicating higher IJDs.

*Burnout* was measured at T1 and T2, using a nine-item version of the Bergen Burnout Inventory (BBI-9; Salmela-Aro et al., 2011; see also Feldt et al., 2014). BBI-9 measures exhaustion with 3 items, e.g., ‘I often sleep poorly because of the circumstances at work’ (*M* = 3.07, *SD* = 1.15, *α* = 0.74); cynicism with 3 items, e.g., ‘I feel that I have gradually less to give’ (*M* = 2.52, *SD* = 1.17, *α* = 0.82); and inadequacy with 3 items, e.g., ‘My expectations to my job and to my performance have reduced’ (*M* = 2.78, *SD* = 1.25, *α* = 0.75). Participants responded to each item on a 6-point Likert-type scale (where 1 = totally disagree and 6 = totally agree). From these, the mean scores were then calculated for the three dimensions of burnout, with higher scores indicating higher burnout.

*Affective-identity motivation to lead (AI-MTL)* was measured using the Motivation to Lead Questionnaire (Chan and Drasgow, 2001) at T1. The five items for the present study were taken from Bobbio and Rattazzi’s (2006) shortened version of the MTL questionnaire, e.g., ‘most of the time I prefer being a leader rather than a follower when working in a group’ (*M* = 3.09, *SD* = 0.75, *α* = 0.81). Participants responded to each item on a 5-point Likert scale (where 1 = totally disagree and 5 = totally agree). From these the mean score was calculated (two items reversed), with higher scores indicating higher AI-MTL.

*Leadership status* was measured using a dichotomous variable. If participants maintained their professional position without leadership duties during the follow-up it was coded as 0, and 1 if they occupied a leadership position.

*Control variables* included gender (female = 0, male = 1), age (years), length of working week (in hours), and occupational background (no = 0, yes = 1, for professors, other university academics and researchers, business school graduates, technical academics). The baseline of the dependent variable was taken into account by controlling for measures of burnout at T1. In spite of inconsistent evidence, gender and age were included because some studies (e.g., Rollero et al., 2016; Zacher and Schmitt, 2016) suggest that these variables be associated with occupational well-being. Working hours were similarly included since adverse health consequences suggested to follow overly long weekly working hours (e.g., Geurts et al., 2014; Kivimäki et al., 2015). Descriptive information about the study variables is summarized in Table 1.

**Table 1:** Correlations for the study variables.

Variable	1	2	3	4	5	6	7	8	9	10	11	12	13
1. IJPDs^a^ T1	–												
2. ICPDs^a^ T1	0.57^***^	–											
3. ILDs^a^ T1	0.46^***^	0.30^***^	–										
4. Exhaustion^a^ T1	0.22^***^	0.22^***^	0.25^***^	–									
5. Cynicism^a^ T1	0.05	0.16^**^	−0.03	0.36^***^	–								
6. Inadequacy^a^ T1	0.11^*^	0.21^***^	−0.09	0.37***	0.77***	–							
7. Exhaustion^a^ T2	0.17^***^	0.20^***^	0.22^***^	0.64^***^	0.15^**^	0.20^***^	–						
8. Cynicism^a^ T2	0.08	0.09	0.05	0.28^***^	0.48^***^	0.41^***^	0.39^***^	–					
9. Inadequacy^a^ T2	0.12^*^	0.17^**^	0.06	0.28^***^	0.41^***^	0.51^***^	0.32^***^	0.75^***^	–				
10. AI-MTL^a^ T1	0.09	0.24^***^	−0.08	0.06	−0.02	0.03	0.00	−0.13^*^	−0.02	–			
11. Age^a^	0.05	−0.07	0.15^**^	0.09	0.02	0.06	0.08	0.03	0.02	−0.05	–		
12. Length of working week^a^	0.08	0.06	−0.02	0.30^***^	−0.05	−0.01	0.25^***^	0.04	0.03	0.13^*^	0.08	–	
13. Gender^b,1^	−0.04	−0.09	−0.08	−0.17^**^	0.01	0.05	−0.17^**^	0.01	−0.03	−0.09	−0.06	−0.06	–
14. Professors^b,2^	0.03	−0.07	0.03	0.13^*^	0.06	0.05	0.07	0.02	0.02	−0.00	0.16^**^	0.22^***^	0.03
15. Other university academics and researchers^b,2^	0.04	0.13^*^	0.02	0.21^***^	−0.04	0.03	0.21^***^	0.07	0.06	−0.04	0.10	0.17^**^	−0.14^**^
16. Business school graduates^b,2^	0.06	0.03	0.05	−0.11^*^	0.03	−0.02	−0.11^*^	−0.08	−0.01	0.07	−0.19^***^	−0.10^*^	−0.08
17. Technical academics^b,2^	−0.11^*^	−0.15^**^	−0.09	−0.19^***^	−0.01	−0.04	−0.18^***^	−0.02	−0.06	−0.02	−0.01	−0.20^***^	0.23^***^

*p < 0.05, **p < 0.01, ***p < 0.001. Significance tests are two-tailed.

^a^Pearson, ^b^Spearman.

IJPDs, Intensified job-related planning demands; ICPDs, Intensified career-related planning demands; ILDs, Intensified learning demands; AI-MTL, Affective-identity motivation to lead.

^1^Gender (female = 0, male = 1).

^2^Occupational background (no = 0, yes = 1, for professors, other university academics and researchers, business school graduates, and technical academics).

### 3.3. Statistical analyses

IBM’s SPSS Statistics 27 software was used in all the statistical analyzes. First the intercorrelations among the main and background variables were explored using either Spearman’s or Pearson’s correlations (depending on the scale of the variable), and the statistically significant background variables were then set as covariates. We then used hierarchical linear modeling (see Baron and Kenny, 1986; Dawson, 2014) to investigate the longitudinal associations between IJDs and later burnout. Two-way interaction terms (dimension of IJDs at T1 × AI-MTL at T1) were formed to study the moderating role of AI-MTL. Three-way interaction terms (dimension of IJDs at T1 × AI-MTL at T1 × leadership status at T2) were formed to study whether the effects differ between those who had become leaders and those who had not. A significant three-way interaction would indicate that the moderating effect of AI-MTL is dependent on one’s leadership status in the follow-up.

All variables –except for the dependent variable –were standardized before entering them in the model and each categorical variable was given a code (0, 1) for consistency (Dawson, 2014). Seven steps were then taken to see the extent to which the IJPD, ICPD, and ILD dimensions of IJDs at T1 could each relate to burnout in terms of exhaustion, cynicism, and inadequacy at T2. First (i) the baseline of the dependent variable was entered (burnout at T1); then (ii) possible control variables; then (iii) the particular independent variable (dimension of IJDs at T1); then (iv) the first moderator variable (AI-MTL at T1); then (v) the second moderator variable (leadership status at T2). Finally, the interaction terms were entered: (vi) two-way (dimension of IJDs at T1 x AI-MTL at T1, dimension of IJDs at T1 x leadership status at T2, AI-MTL at T1 × leadership status at T2); and (vii) three-way (dimension of IJDs at T1 × AI-MTL at T1 × leadership status at T2). Altogether, nine regression analyzes were conducted (results reported in Tables 2–4).

**Table 2:** The longitudinal associations between intensified job-related planning demands (IJPDs) and burnout (exhaustion, cynicism, and inadequacy) moderated by affective-identity motivation to lead (AI-MTL) and leadership status.

	Exhaustion T2^a^			Cynicism T2^b^			Inadequacy T2^b^		
	*B*	*R* ^2 adj^	Δ*R*^2^	*B*	*R* ^2 adj^	Δ*R*^2^	*B*	*R* ^2 adj^	Δ*R*^2^
*Baseline of dependent variable*		0.411	0.413^***^		0.224	0.227^***^		0.253	0.255^***^
Burnout dimension T1	0.683 ^***^			0.559^***^			0.633^***^		
*Control variables*		0.416	0.013		–	–		–	–
Gender	−0.161			–			–		
Length of working week	0.039			–			–		
Professors^1^	−0.032			–			–		
Other university academics and researchers^1^	0.163			–			–		
Business school graduates^1^	0.014			–			–		
*Independent variable*		0.416	0.001		0.225	0.003		0.257	0.006
IJPDs T1	0.049			0.067			0.110		
*1. Moderator variable*		0.417	0.003		0.238	0.014^**^		0.256	0.001
AI-MTL T1	−0.053			−0.149^*^			−0.047		
*2. Moderator variable*		0.423	0.008*		0.236	0.001		0.260	0.006
Leadership status T2	0.389^**^			−0.134			−0.318		
*Two-way interaction terms*		0.427	0.009		0.247	0.017^*^		0.278	0.023^**^
AI-MTL × IJPDs	0.060			0.134^**^			0.170^**^		
IJPDs × Leadership status	−0.137			0.025			−0.060		
AI-MTL × Leadership status	−0.245			0.099			0.153		
*Three-way interaction term*		0.425	0.000		0.247	0.002		0.280	0.005
AI-MTL × IJPDs × Leadership status	0.040			−0.150			−0.254		

^*^p < 0.05, ^**^p < 0.01, ^***^p < 0.001. Leadership status at T2 (has maintained a professional position without leadership duties = 0, has occupied a leadership position during follow-up = 1). ^1^Occupational background (no = 0, yes = 1, for professors, other university academics and researchers, business school graduates, and technical academics). Unstandardized *B* is reported here because the predictor and moderator variables were centered before conducting the analyzes. In case of ^a^non-significant interactions, Unstandardized *B* is reported from the final step of the model. In case of ^b^significant two-way interactions, Unstandardized *B* is reported from the second final step, however, for a three-way interaction, Unstandardized *B* is reported from the final step.

**Table 3:** The longitudinal associations between intensified career-related planning demands (ICPDs) and burnout (exhaustion, cynicism, and inadequacy) moderated by affective-identity motivation to lead (AI-MTL) and leadership status.

	Exhaustion T2^c^			Cynicism T2^a^			Inadequacy T2^b^		
	*B*	*R* ^2 adj.^	Δ*R*^2^	*B*	*R* ^2 adj^	Δ*R*^2^	*B*	*R* ^2 adj.^	Δ*R*^2^
*Baseline of dependent variable*		0.411	0.413^***^		0.224	0.227^***^		0.253	0.255^***^
Burnout dimension T1	0.671^***^			0.532^***^			0.592^***^		
*Control variables*		0.416	0.013		–	–		–	–
Gender	−0.157			–			–		
Length of working week	0.054			–			–		
Professors^1^	0.065			–			–		
Other university academics and researchers^1^	0.139			–			–		
Business school graduates^1^	−0.010			–			–		
*Independent variable*		0.418	0.003		0.223	0.001		0.257	0.006
ICPDs T1	0.066			0.049			0.107		
*1. Moderator variable*		0.421	0.004		0.236	0.016^**^		0.257	0.002
AI–MTL T1	−0.061			−0.165^**^			−0.073		
*2. Moderator variable*		0.426	0.007^*^		0.235	0.001		0.262	0.006
Leadership status T2	0.367^**^			−0.132			−0.340^*^		
*Two-way interaction terms*		0.427	0.005		0.241	0.012		0.275	0.020^*^
AI-MTL × ICPDs	−0.016			0.085			0.144^*^		
ICPDs × Leadership status	−0.036			0.107			0.160		
AI-MTL × Leadership status	−0.662 ^**^			−0.171			0.029		
*Three-way interaction term*		0.435	0.010^*^		0.241	0.002		0.276	0.002
AI-MTL × ICPDs × Leadership status	0.510^*^			0.247			0.267		

^*^p < 0.05, ^**^p < 0.01, ^***^p < 0.001. Leadership status at T2 (has maintained a professional position without leadership duties = 0, has occupied a leadership position during follow-up = 1). ^1^Occupational background (no = 0, yes = 1, for professors, other university academics and researchers, business school graduates, technical academics). Unstandardized *B* is reported here because the predictor and moderator variables were centered before conducting the analyzes. In case of ^a^non-significant interactions, Unstandardized *B* is reported from the final step of the model. In case of ^b^significant two-way interactions, Unstandardized *B* is reported from the second final step, however, for a three-way interaction, Unstandardized *B* is reported from the final step. In case of ^c^significant three-way interactions, Unstandardized *B* is reported from the final step of the model.

**Table 4:** The longitudinal associations between intensified learning demands (ILDs) and burnout (exhaustion, cynicism, and inadequacy) moderated by affective-identity motivation to lead (AI-MTL) and leadership status.

	Exhaustion T2^a^			Cynicism T2^a^			Inadequacy T2^a^		
	*B*	*R* ^2 adj.^	Δ*R*^2^	*B*	*R* ^2 adj.^	Δ*R*^2^	*B*	*R* ^2 adj.^	Δ*R*^2^
*Baseline of dependent variable*		0.411	0.413^***^		0.224	0.227^***^		0.253	0.255^***^
Burnout dimension T1	0.658^***^			0.554^***^			0.652^***^		
*Control variables*		0.416	0.013		–	–		–	–
Gender	−0.176			–			–		
Length of working week	0.050			–			–		
Professors^1^	−0.045			–			-		
Other university academics and researchers^1^	0.138			–			–		
Business school graduates^1^	−0.004			–			–		
*Independent variable*		0.421	0.006		0.227	0.005		0.262	0.011^*^
ILDs T1	0.072			0.070			0.150^*^		
*1. Moderator variable*		0.421	0.002		0.237	0.012^*^		0.261	0.000
AI-MTL T1	−0.035			−0.142^*^			−0.030		
*2. Moderator variable*		0.427	0.007^*^		0.236	0.001		0.265	0.006
Leadership status T2	0.342^*^			−0.161			−0.349^*^		
*Two-way interaction terms*		0.431	0.009		0.231	0.002		0.267	0.008
AI-MTL × ILDs	−0.065			0.012			0.105		
ILDs × Leadership status	0.167			0.160			−0.010		
AI-MTL × Leadership status	−0.208			0.267			0.300		
*Three-way interaction term*		0.430	0.000		0.235	0.007		0.271	0.007
AI-MTL × ILDs × Leadership status	−0.052			−0.334			−0.362		

^*^p < 0.05, ^**^p < 0.01, ^***^p < 0.001. Leadership status at T2 (has maintained a professional position without leadership duties = 0, has occupied a leadership position during follow-up = 1). ^1^Occupational background (no = 0, yes = 1, for professors, other university academics and researchers, business school graduates, and technical academics). Unstandardized B is reported here because the predictor and moderator variables were centered before conducting the analyzes. In case of ^a^non-significant interactions, Unstandardized *B* is reported from the final step of the model.

**Figure 1:**
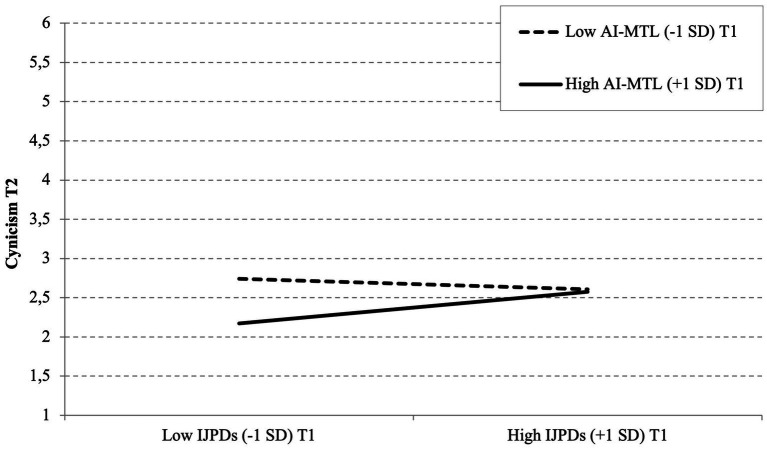
Two-way interaction effect between affective-identity motivation to lead (AI-MTL) at T1 and intensified job-related planning demands (IJPDs) at T1 on cynicism at T2 (*B* coefficients are reported in the text).

**Figure 2:**
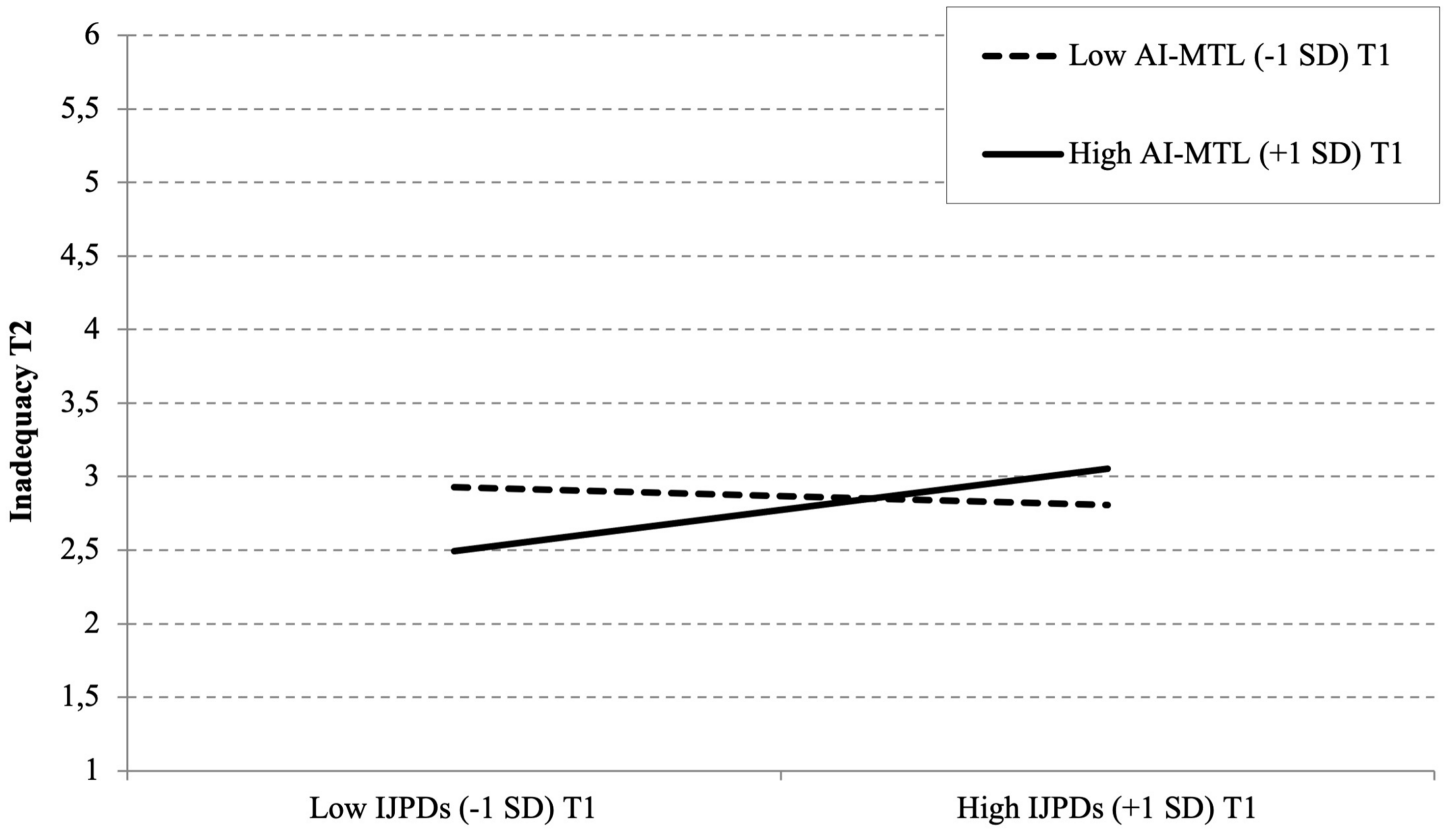
Two-way interaction effect between affective-identity motivation to lead (AI-MTL) at T1 and intensified job-related planning demands (IJPDs) at T1 on inadequacy at T2 (*B* coefficients are reported in the text).

**Figure 3:**
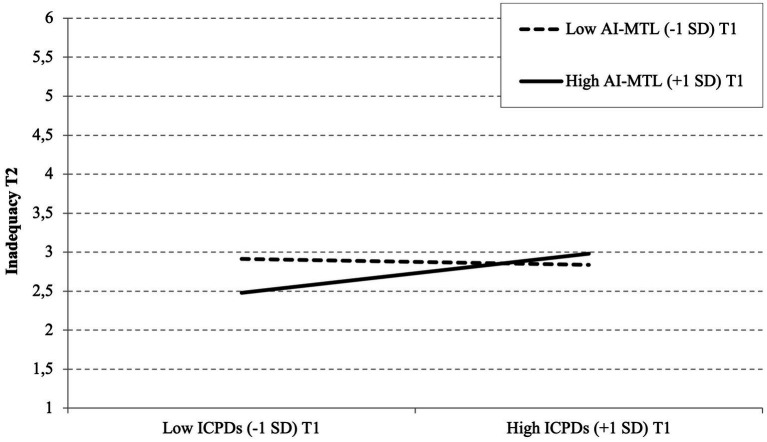
Two-way interaction effect between affective-identity motivation to lead (AI-MTL) at T1 and intensified career-related planning demands (ICPDs) at T1 on inadequacy at T2 (*B* coefficients are reported in the text).

**Figure 4:**
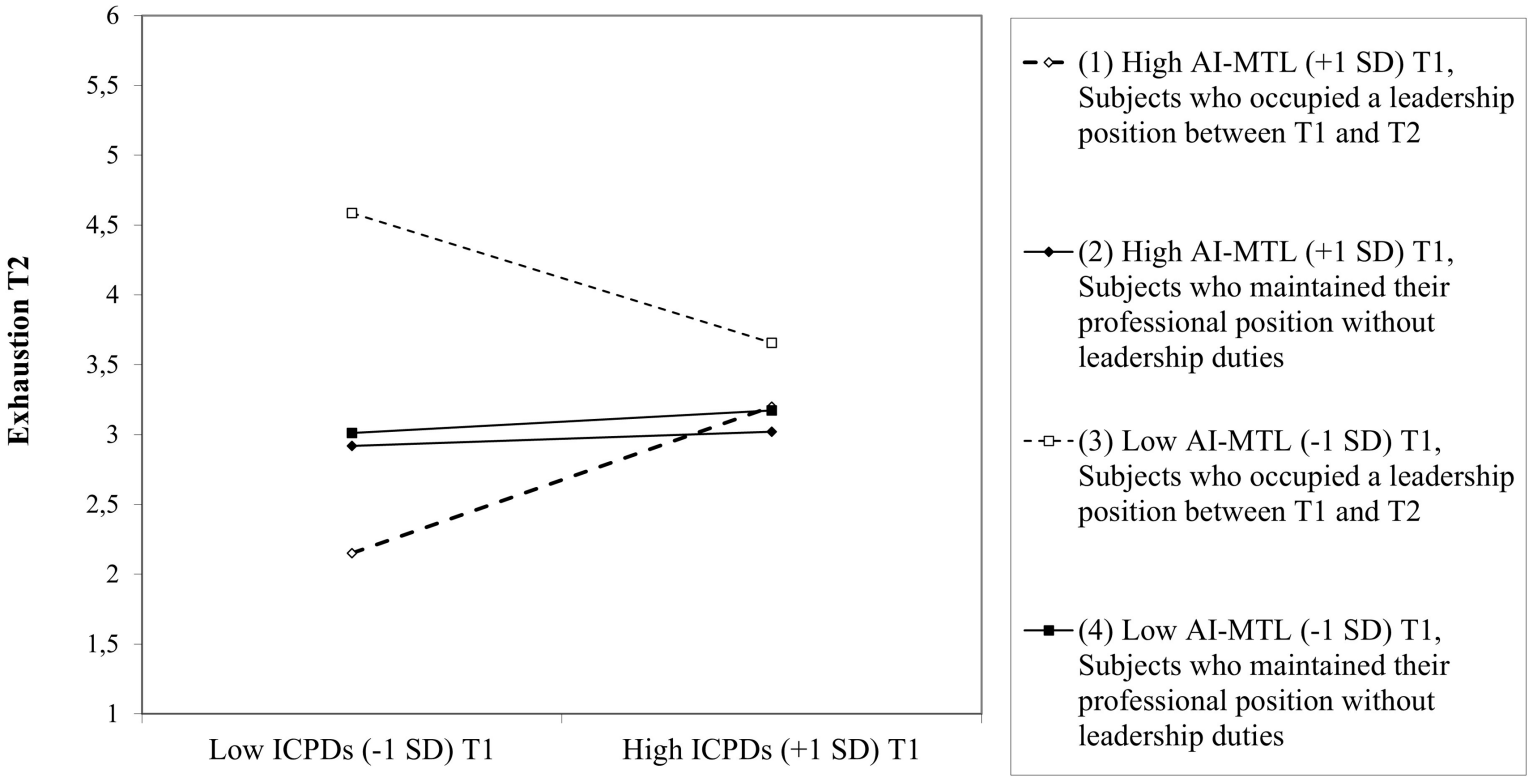
Three-way interaction effect between affective-identity motivation to lead (AI-MTL) at T1, intensified career-related planning demands (ICPDs) at T1 and leadership status on exhaustion at T2 (*B* coefficients are reported in the text).

All significant two-way interactions then underwent a simple slope analysis to test their significance under high, median, and low (−1 SD, Mean, +1 SD) scores of the moderator variable (Dawson, 2014). In three-way interactions that were significant, a simple slope analysis was performed to test their significance under low (−1 SD) and high (+1 SD) scores of moderator variable (AI-MTL) and two options of leadership status (dichotomous moderator variable: maintaining professional position without leadership duties during follow-up / occupying a leadership position during follow-up). The results of simple slope analyzes are seen in Figures 1–4.

## 4. Results

### 4.1. Descriptive results

Based on correlational analysis (Table 1), the burnout dimensions showed significant correlations over time (test–retest *r =* 0.48–0.64) indicating that burnout remained relatively stable over 2 years. While all three IJD dimensions measured at the study baseline had significant positive correlations with exhaustion 2 years later, only IJPDs and ICPDs had such a correlation with inadequacy 2 years later. Meanwhile, there was significant negative correlation between AI-MTL at the study baseline and cynicism (a higher AI-MTL indicated lower cynicism 2 years later); and significant positive correlations between AI-MTL and ICPDs on the one hand, and length of working week on the other (both at study baselines). The background variables only correlated with exhaustion 2 years later; the number of working hours per week correlated positively, as did being a woman (gender) and being a professor or other university academic/researcher (occupation), while business school graduates and technical academics were more likely to report lower levels of exhaustion 2 years later.

### 4.2. Intensified job-related planning demands (IJPDs) and burnout

As shown in Table 2, IJPDs were not related to any of the burnout dimensions so *H1a* was not supported. For the two-way interactions, AI-MTL did moderate the association between IJPDs and two of the dimensions of burnout – cynicism and inadequacy – but not exhaustion. However, the significant moderator effects were not consistent with *H2a* as shown graphically in Figures 1, 2. *H3a* was not supported either as AI-MTL functioned similarly regardless of leadership status 2 years later (i.e., three-way interaction effects on burnout dimensions were not significant).

According to a simple slope analysis (see Figure 1), there was a significant positive association between IJPDs and cynicism (*B* = 0.206, *p* = 0.008) under the condition of high AI-MTL. The same association was only slightly positive under the condition of median AI-MTL (*B* = 0.067) and slightly negative (*B* = −0.072) under the condition of low AI-MTL, however these last two associations were not significant (*p* = 0.222 and *p* = 0.353, respectively). In other words, having high AI-MTL strengthens the association between IJPDs and cynicism, regardless of leadership status at T2. Overall, when IJPDs were low, high level of AI-MTL associated with lower levels of cynicism compared to when the level of AI-MTL was low, but when IJPDs were high, there was no difference.

Because AI-MTL was found to have a similar moderation effect on feelings of inadequacy at T2, it also underwent a simple slope analysis (see Figure 2) which showed that there was a significant positive relationship between IJPDs and inadequacy under the condition of high AI-MTL (*B* = 0.259, *p* < 0.001). As with cynicism, no significant associations were found between IJPDs and inadequacy under conditions of either median or low AI-MTL (*B* = 0.089, *B* = −0.081, and *p* = 0.105, *p* = 0.296, respectively). In other words, having high AI-MTL strengthens the association between IJPDs and inadequacy, regardless of leadership status at T2. Overall, when IJPDs were low, high AI-MTL was associated with lower levels of inadequacy. In turn, when IJPDs were high, high AI-MTL was linked with higher levels of inadequacy.

### 4.3. Intensified career-related planning demands (ICPDs) and burnout

As seen in Table 3, ICPDs were not related to any of the burnout dimensions so *H1b* was not supported; and neither was *H2b* because, even if AI-MTL moderated the relationship of ICPDs with inadequacy (though not exhaustion and cynicism), this relationship was positive – i.e., not negative as we had hypothesized. Simple slope analysis (see Figure 3) confirmed a significant positive relationship between ICPDs and inadequacy under the condition of high AI-MTL and – to a lesser extent – under the condition of median AI-MTL (*B* = 0.275, *B* = 0.122 and *p* < 0.001, *p* = 0.027, respectively). Although the moderated association was slightly negative under the condition of low AI-MTL, it was not significant (*B* = −0.031, *p* = 0.689). The results indicate that the higher the AI-MTL is, the stronger the association between ICPDs and inadequacy, regardless of leadership status at T2. Overall, under conditions of low ICPDs, high AI-MTL was associated with lower levels of inadequacy. In turn, when ICPDs were high, high AI-MTL was associated with higher levels of inadequacy.

Although significant three-way interaction (AI-MTL x ICPDs x leadership status) was found for exhaustion indicating differences regarding leadership status (see Table 3), AI-MTL appeared to have no buffering effect, so H3b was also rejected. According to simple slope analysis (see Figure 4), the relationship between ICPDs and exhaustion was significantly positive (*B* = 0.524, *p* = 009) under the condition of high AI-MTL and leadership position. In other words, high AI-MTL strengthens the connection of ICPDs to later exhaustion in those subjects who had occupied a leadership position during the follow-up period. In turn, the relationship between ICPDs and exhaustion was negative under the condition of low AI-MTL and occupied leadership position, but the coefficient was non-significant (*B* = −0.464, *p* = 0.081). No significant associations were found between ICPDs and exhaustion under conditions of high or low AI-MTL and professional position without leadership status (high AI-MTL: *B* = 0.050, *p* = 0.489, and low MTL: *B* = 0.082, *p* = 0.237). In other words, having high (or low) AI-MTL has no role in the relationship between IJPDs and later exhaustion among those subjects who maintained their professional position without leadership duties during follow-up.

### 4.4. Intensified learning demands (ILDs) and burnout

The results of regression analyzes (Table 4) showed that the only dimension of IJDs to associate with burnout in the follow-up was ILDs. Greater ILDs associated with feelings of greater inadequacy 2 years later, but because no associations with exhaustion or cynicism were found, *H1c* could only be partially supported. Further, neither *H2c* nor *H3c* could be supported as AI-MTL did not moderate the relationship between ILDs and any dimension of burnout.

## 5. Discussion

The main objective of this longitudinal study was to increase our understanding of ways to support sustainable careers among highly educated professionals who, in the face of intensifying work demands, either occupy or do not take on a leadership position. We approached it by looking at whether IJDs are related to burnout 2 years later, and whether AI-MTL moderates this relationship and thereby functions as a personal resource, buffering against the negative effects of IJDs. In addition, we investigated whether this moderating effect might be stronger among those who occupied a leadership position during the follow-up than those who did not.

### 5.1. How intensified learning demands (ILDs) correspond with inadequacy 2 years later

ILDs were the only dimension of the three IJDs to associate with subsequent burnout in the follow-up. More closely, greater ILDs were related to stronger feelings of inadequacy 2 years later, but not feelings of greater exhaustion or cynicism. The association with inadequacy could have occurred because ILDs are about increased requirements to update one’s expertise or adopt new work processes (Kubicek et al., 2015) while inadequacy covers feelings of lack of achievement and competence on the job (Maslach et al., 2001). Thus, the pressure to meet ILDs may risk one’s self-esteem related to feelings of competence whereas exhaustion and cynicism would not be that easily evoked by ILDs. ILDs may not necessarily lead to exhaustion if the job is only moderately straining in other aspects. Learning new skills and knowledge may be motivating as well (challenge demands; LePine et al., 2005), which again may act as a counterforce for cynicism.

Interestingly, neither IJPDs nor ICPDs were found to be associated with any of the burnout dimensions. This finding is not in line with previous studies, which have shown that job demands are generally likely to trigger health-impairment processes (Bakker and Demerouti, 2017; Mazzola and Disselhorst, 2019), and that all IJDs relate to higher total burnout (Mauno et al., 2019). One reason for our finding could be that we studied highly educated professionals (e.g., academics), for whom the freedom to independently plan their jobs and careers might well have been an important factor in gravitating toward their field in the beginning. As such, they would have been more predisposed toward dealing with intensified job demands – as sources of both empowerment and strain – thus not so clearly detrimental to well-being.

Another reason for our findings could be previous studies (Bakker and Demerouti, 2017; Mazzola and Disselhorst, 2019) investigating rather different types of job demands. Not only this, but whereas Mauno et al. (2019) measured burnout in total, our study went into more detail, differentiating between three dimensions of burnout. Interestingly, our finding that IJDs did not associate with cynicism tallied with Huhtala et al. (2021) but whereas they found IJDs to associate with exhaustion, we did not. The reason for this could be the difference in work context (i.e., healthcare; Huhtala et al., 2021), where intensified demands would be heightened. However, we must bear in mind that the overall amount of previous research on IJDs remains scarce (Mauno et al., 2019), so our understanding of the consequences of different IJDs are far from complete.

### 5.2. Affective-identity motivation to lead (AI-MTL) may strengthen the negative effects of intensified demands on burnout

Contrary to our expectations, AI-MTL strengthened the negative burnout effects of IJDs rather than acting as a buffer. Even though the buffer role was not found, it is worth noticing that when IJPDs and ICPDs are low, burnout seems to be lower among participants with high AI-MTL. Among our sample in which 83% of participants maintained their professional positions without taking on formal leadership duties, this finding gives a reason to wonder if AI-MTL could be beneficial also in roles without a formal leader status. On the other hand, our results might also reflect person-career misfit: When IJDs are high, and particularly when they are related to job and career planning, a professional with high AI-MTL might realize that their present position does not really fit with their character, values and goals (i.e., identity). This again might lead to a sense of meaninglessness and trigger symptoms of burnout.

AI-MTL strengthened the association between IJPDs and feelings of cynicism and inadequacy, regardless of leadership status. Thus, it can be concluded that when investigated professionals felt they were increasingly required to organize their daily work independently (Kubicek et al., 2015), high AI-MTL made them more vulnerable to feeling cynical and/or inadequate. Among professionals who do not have formal leadership duties, yet who have high AI-MTL, this cynicism (i.e., negative attitude toward work; Maslach et al., 2001) may come from feelings of ‘having less to offer’ (Salmela-Aro et al., 2011) as AI-MTL is based on the idea that one contributes more as a leader than as a follower (Chan and Drasgow, 2001). Equally, inadequacy may be the result of feeling less competent (Maslach et al., 2001). High AI-MTL may thus actually endanger well-being and sustainable careers when job-related planning and decision-making demands intensify.

AI-MTL was found to have a similar strengthening effect on the association between ICPDs and feelings of inadequacy. Even moderate levels of AI-MTL were found to have this effect, but to a lesser extent than among those reporting high AI-MTL. Again, among professionals, this could result from realizing that they are not in a position where they could utilize their AI-MTL, which again may lead to frequently questioning the value of one’s work (Salmela-Aro et al., 2011) –especially in an environment where employees are increasingly responsible for creating networks and for ensuring their own personal career development (Kubicek et al., 2015). High job demands could also highlight the consequences of decisions made earlier in a career, thereby endangering feelings of well-being.

One could reasonably wonder from the above results if AI-MTL might function as a useful personal resource for highly educated professionals – without formal leadership duties – in situations where job demands are not highly intensified. However, if these demands intensify, the role of work meaningfulness and value-based actions (i.e., person-career fit) could be emphasized; coping with intensified demands might be harder if the work itself does not allow contributing others and expressing the true identity of oneself (Martela and Pessi, 2018).

### 5.3. Differences in affective-identity motivation to Lead (AI-MTL) according to leadership status

We found some differences according to leadership status: high AI-MTL strengthened the association between ICPDs and exhaustion only among those subjects who had become leaders during follow-up - the association with exhaustion was not observed among non-leaders with high AI-MTL. This finding could be explained, for example, by agentic orientation which is thought to be stronger among people reporting higher AI-MTL (Badura et al., 2020). Agentic orientation is related to proactivity which has been suggested to help in regulating strain (Bakker and De Vries, 2021), however, in addition to proactivity, it is linked with an emphasis on achieving (Eagly and Karau, 2002). Hence, leaders with high AI-MTL could be threatening their occupational well-being by being highly self-demanding as they may put more emphasis on achieving higher career-related demands compared to leaders with low AI-MTL.

Another such difference was that leaders with high AI-MTL reported less exhaustion at the end of the follow-up compared to leaders with low AI-MTL – regardless of the level of ICPDs at the study baseline; among the whole sample, of which the majority did not take on a leadership position by T2, participants with high AI-MTL reported better occupational well-being only if ICPDs were low. Although AI-MTL marginally strengthened the association between intensified career-related demands and exhaustion, it still seems that AI-MTL (i.e., intrinsic motivation toward leadership) functions, to some extent, as a personal resource for leaders to fight exhaustion. This supports previous findings about AI-MTL as a resource for leaders (Auvinen et al., 2020, 2021). Leaders with high AI-MTL are more likely to experience leadership self-efficacy (Chan and Drasgow, 2001; Badura et al., 2020) which may enhance the experience of controlling the overall picture. Thus, it may help in coping with the more stressful aspects of work – and in dealing with particularly high demands associated with leadership positions (Skakon et al., 2011; Li et al., 2018).

### 5.4. Theoretical contributions and practical implications

This study contributes to existing literature on sustainable careers and MTL. To the best of our knowledge, it is the first longitudinal study on how AI-MTL affects the relationship between intensified job demands and burnout among highly educated professionals, some of whom occupied a leadership position during the follow-up. The resource perspective of AI-MTL has been previously studied cross-sectionally and only among leaders (Auvinen et al., 2020), whereas our study is longitudinal and 83% of our sample were not in formal positions of leadership. Moreover, IJPDs, ICPDs, and ILDs are central from the career perspective and have, thus far, been of only minor research interest – particularly among longitudinal studies. Overall, we broadened the concept of person-career fit by exploring whether MTL would also be beneficial for those outside formal leadership roles (i.e., among highly educated professionals).

Our findings provide valuable insights for human resource management. In an era of accelerating digitalization (Rosa, 2003, 2013), the association between ILDs and feelings of inadequacy within an organization need to be recognized: for example, when developing and implementing new technologies and when motivating employees to adopt new knowledge and skills. In other words, if learning demands are high, the support offered should be high as well. Moreover, in order to promote sustainable careers in the midst of these intensified demands, it might be beneficial to consider the level of AI-MTL –in addition to other factors –when recruiting new professionals. Regardless of their current leadership status, it might be beneficial to discuss with them about matters that relate to their AI-MTL –what kind of positive effects it might have for one’s occupational well-being but also what kind of risks it might be linked with in that specific position. The discussion could aim to utilize one’s motivational resources without sacrificing one’s well-being when the demands increase.

### 5.5. Limitations and further research

Our study nevertheless has some limitations that must be taken into consideration. To begin with, the variables we studied were measured using self-reports. In future studies on this topic, questionnaires could be complemented by other data collection methods such as interviews or a health tracking system for assessing burnout-related symptoms. Studying changes over a longer period of time would also have allowed us to uncover more detailed results on leaders as they would have occupied their leadership positions for a longer time. New challenges followed by a position change might intensify the overall job demands experienced and the effect of this change on exhaustion may thus well be temporary. Additionally, the concept of AI-MTL was originally utilized to detect the suitable candidates to fill the formal leadership roles (Chan and Drasgow, 2001) and therefore it may not tap onto, for example, the self-leadership and shared leadership features of professional work properly. However, considering also the possibility to occupy leader roles that many professionals face, it was reasonable to study a motivational construct related to more formal leader roles.

The generalizability of this study is also limited as the results are, to a certain extent, sample-specific: we studied a group of highly educated Finnish professionals; and, although the size of the sample was satisfactory (*n* = 372), the number of participants in leadership positions was relatively small (*n* = 63). Therefore, this study should be replicated applying larger groups and more evenly matched group sizes.

Although the importance of AI-MTL is highlighted by most studies (Badura et al., 2020), we would have gathered more detailed and contrastive information if all the dimensions of leader motivation had been studied simultaneously. MTL dimensions have been found in different combinations (i.e., profiles) among leaders (Auvinen et al., 2020) and so the buffering role of AI-MTL might be better understood by considering different combinations of MTL dimensions as moderators. Finally, to broaden the literature of AI-MTL as a potential resource for all professionals, regardless of their leadership status, career sustainability should be investigated by other indicators than just burnout.

## 6. Conclusion

This study has contributed to the literature on the motivation to lead, building on the previous research of AI-MTL in the context of sustainable careers and resources (Auvinen et al., 2020, 2021). We found that intensified learning demands at work may be associated with feelings of inadequacy 2 years later. Although the results regarding AI-MTL as a buffer did not turn out as hypothesized, this study broadened the perspective of person-career fit by investigating AI-MTL as a personal resource for all professionals, regardless of their leadership status. It seems that for leaders, AI-MTL could function as a resource for tackling exhaustion specifically –no matter the intensity of job demands. For professionals without formal leadership duties, it could help tackle cynicism and inadequacy when job demands are not highly intensified. However, we cannot ignore that AI-MTL in professionals may also lead to increased vulnerability by capturing elements that, in excess, strengthen the association between intensified job demands and burnout. Perhaps there is an optimal level of AI-MTL that would help to ensure occupational well-being and sustainable careers. Occupational well-being is yet not only a responsibility of an individual but also structural and societal actions are needed. This study is a part of this conversation, highlighting the aspects that could help build meaningfulness in professional and managerial work, that serves as an important resource for lasting, enduring careers.

## Data availability statement

The datasets presented in this article are not readily available because anonymized data is not transferred outside the EU/EEA area. Requests to access the datasets should be directed to, taru.feldt@jyu.fi.

## Ethics statement

Ethical review and approval was not required for the study on human participants in accordance with the local legislation and institutional requirements. The patients/participants provided their written informed consent to participate in this study.

## Author contributions

KL and AT shared the first authorship, contributed equally to this work, performed the statistical analysis, and drafted the manuscript. EA, MH, and TF provided comments to the manuscript and were involved in the data collection. TF acted as a principal investigator (PI) in the study project. All authors conceived the study design, contributed to the article, and approved the submitted version.

## Funding

The study was supported by the Finnish Work Environment Fund (project 200320; PI TF) and Academy of Finland (project 308336; PI TF).

## Conflict of interest

The authors declare that the research was conducted in the absence of any commercial or financial relationships that could be construed as a potential conflict of interest.

## Publisher’s note

All claims expressed in this article are solely those of the authors and do not necessarily represent those of their affiliated organizations, or those of the publisher, the editors and the reviewers. Any product that may be evaluated in this article, or claim that may be made by its manufacturer, is not guaranteed or endorsed by the publisher.
